# Novel Flexible Wearable Sensor Materials and Signal Processing for Vital Sign and Human Activity Monitoring

**DOI:** 10.3390/s17071622

**Published:** 2017-07-13

**Authors:** Amir Servati, Liang Zou, Z. Jane Wang, Frank Ko, Peyman Servati

**Affiliations:** 1 Department of Electrical and Computer Engineering, University of British Columbia, Vancouver, BC V6T 1Z4, Canada; liangzou@ece.ubc.ca (L.Z.); zjanew@ece.ubc.ca (Z.J.W.); 2 Department of Materials Engineering, University of British Columbia, Vancouver, BC V6T 1Z4, Canada; frank.ko@ubc.ca

**Keywords:** wearable devices, smart textile, health monitoring, flexible electronics, signal processing, nanomaterials

## Abstract

Advances in flexible electronic materials and smart textile, along with broad availability of smart phones, cloud and wireless systems have empowered the wearable technologies for significant impact on future of digital and personalized healthcare as well as consumer electronics. However, challenges related to lack of accuracy, reliability, high power consumption, rigid or bulky form factor and difficulty in interpretation of data have limited their wide-scale application in these potential areas. As an important solution to these challenges, we present latest advances in novel flexible electronic materials and sensors that enable comfortable and conformable body interaction and potential for invisible integration within daily apparel. Advances in novel flexible materials and sensors are described for wearable monitoring of human vital signs including, body temperature, respiratory rate and heart rate, muscle movements and activity. We then present advances in signal processing focusing on motion and noise artifact removal, data mining and aspects of sensor fusion relevant to future clinical applications of wearable technology.

## 1. Introduction

The novel field of wearable technology is changing rapidly in recent years and has become an important contender in consumer electronic market. Currently, the global wearable market is worth about $30 billion US dollars and is estimated to grow as much as $100 and $150 billions by 2023 and 2026, respectively [1]. The majority of the available wearable products are in the form of smart watches and fitness bands, which can provide consumers with information about activity, body movements and some consumer use vital signs. Despite these successes, the use of wearable devices in real clinical applications has been limited, mainly due to their limited accuracy, validity and reliability [2,3]. In addition, the bulky and non-flexible nature of existing devices, limit the form factor and duration of comfortable use. Moreover, high power consumption of the sensors and data processing and analytic hardware puts constraints long-term operability and forces developers to sacrifice accuracy for increase in the battery life. Other important limitations include the limited anatomical locations used for sensor placement, motion artifacts and handling/interpreting the large volume of generated data [4]. It could be argued that the opportunities in the huge market of digital and personalized healthcare hinder upon addressing these challenges in addition to training and market adaptation.

Sensors are the heart of a wearable monitoring device. One of the main reasons behind the low accuracy, high power consumption and rigidity of today’s wearable device is the property of sensing elements, how they are placed on the body and the need for extensive signal processing to remove noise and extract the desired features. Therefore, development of novel sensors is important to address the previously mentioned challenges. Recent advances in nanomaterials, flexible electronics and smart textiles highlight opportunities to materialize wearable devices with real clinical applications. Novel materials used in these sensors are light, naturally conformable resulting in a better interface with skin, and can provide better signal quality.

In this paper, we review some of the advances in materials and sensing technologies reported in the field of wearable health monitoring, and highlight some of their advantages and disadvantages. Section 2 focuses on the materials and sensors developed for vital sign monitoring, including body temperature, heart rate and respiratory rate. In Section 3, we describe some of the development for better monitoring of human movement, muscle functions and posture. Finally, Section 4 is devoted to the advances in the important field of signal processing and data extraction for wearable devices.

## 2. Vital Sign Monitoring

Accurate monitoring of vital signs is the most critical part of a good clinical assessment. Conventionally, this work has been done by health care professionals at clinics or emergency wards, which provide a sporadic snapshot of the health condition of a patient. Availability of monitoring devices, which can continuously measure vital signs such as heart rate and body temperature, despite inaccuracy and unreliability problems, opens up a huge opportunity for temporal monitoring of patients. Most important vital signs that are commonly monitored by healthcare professionals include body temperature (T), heart rate (HR), respiratory rate (RR) and blood pressure (BP). Monitoring of these vital signs require different measurement modalities and anatomical locations for improved signal quality and accuracy. Table 1 summarizes examples of advanced materials reported for wearable vital sign monitoring, discussed in the following subsections, along with associated features and limitations.

### 2.1. Novel Materials for Body Temperature Measurement

Elevated body temperature is one of the first defense mechanisms of our body in response to illnesses such as infectious diseases. On the other hand, many conditions such as late stage infectious diseases, blood depletion conditions and application of drugs and toxins may lead to a noticeable decrease in body temperature [19,20]. Therefore, continuous monitoring of body temperature could have huge impact on early detection and better management of different diseases and conditions. It is important to mention that, although the core body temperature is maintained tightly at around 37 °C, the surface temperature is slightly lower and depends on parameters including measurement location, body posture, and gender [21,22]. Therefore, proper methods for measurement of surface temperature that accurately estimates core temperature are necessary. The most common locations for wearable temperature sensors are arms and chest. Novel flexible sensors have the potential to improve reliability of the contact of sensor to skin as well as providing more comfort for long-term monitoring.

**Figure 1:**
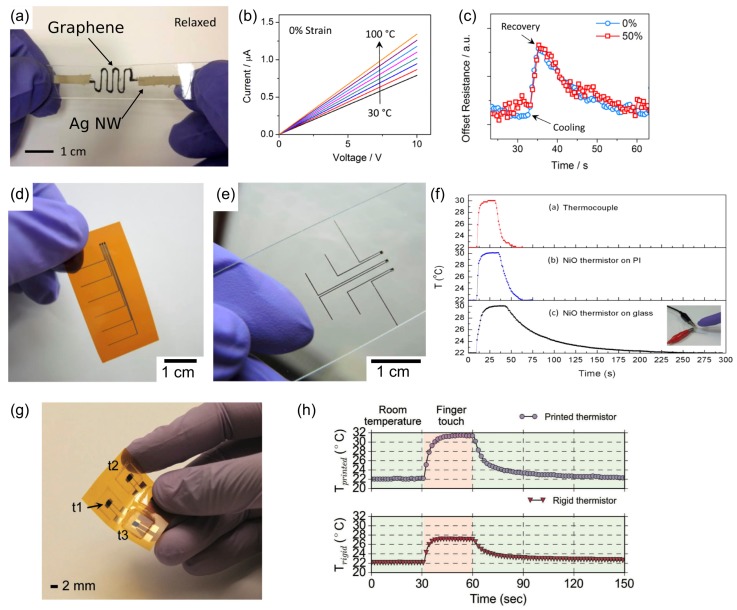
Flexible wearable temperature sensors: (**a**) stretchable graphene thermistor with AgNW (Ag nanowire) electrodes as contacts at relaxed state; (**b**) its I-V curves when temperature changing from 30 to 100 °C; and (**c**) transient response to changing temperature at 0 and 50% strains (Reprinted with permission [5]. Copyright 2015, American Chemical Society); Printed thermistor array on: (**d**) polyimide substrates; and (**e**) on glass; (**f**) Responses of the sensors during touch and leave compared to a thermocouple (Reprinted with permission [6]. Copyright 2013, American Chemical Society); (**g**) Thermistors printed on PI substrate using stencil-printing method, thermistors are labeled as t1, t2, and t3; and (**h**) responses to changing temperature caused by finger touch, compared to commercially available rigid thermistors (Reprinted with permission [7]. Copyright 2016, John Wiley and Sons).

A promising method to develop flexible sensors for temperature monitoring is to use thermistors that operate based on thermoelectric effect [5,6,7,8,23]. The sensitivity of this type of sensor is directly related to the material constant *β* and temperature coefficient *α* of the thermistor. *β* is linearly related to Boltzmann relation $\left(\frac{E}{kT}\right)$, where *E* is bandgap, *k* the Boltzmann’s constant and *T* the temperature of the sensing material. On the other hand, *α* describes the percentage change of resistance per degree Kelvin ($\alpha =-\frac{\beta }{T^{2}}\left(\%K^{-1}\right)$. Yan et al. [5] have reported a flexible temperature sensor based on three-dimensional crumpled graphene as a flexible sensing material and Ag nanowire as electrodes (see Figure 1a). The encapsulated sensor shows a robust performance that is comparable to commercial thermistors (*β* = 835.72 K and *α* = −1.12% K^−1^) even at 50% stretch, although *β* increases under applied strain (Figure 1b,c). Huang et al. [6] reported a thermistor sensor made from inkjet printed NiO arrays on flexible substrate (Figure 1d) or on glass (Figure 1e). The sensors show high sensitivity (*β* ~ 4300 K and *α* = −5.71% K^−1^) and fast response time comparable to a thermocouple (Figure 1f). Khan et al. [7] have also reported similar approach for printing NiO films as thermistor using stencil printing (Figure 1g,h), resulting in high sensitivity (*β* = 4330 K and *α* = −5.84% K^−1^). Recently, Lebedev et al. [8] reported a flexible thermistor based on nanostructure organic semiconductor films of bis(ethylendithio)-tetrathiafulvalene (BEDT-TTF) with potential for integration into textile.

Despite all of these advances in development of a sensitive and efficient wearable temperature sensor, several challenges need to be addressed. One major challenge with all thermistor-based sensors is the sensitivity of the signal to strain and motion [5,6], which is a major issue in wearable devices. One way to overcome this challenge is to have a small rigid thermistor as a reference to correct for the motion effects. This can limit the design and form factor of the device. Another important challenge of wearable temperature sensors is the contact problem, which can lead to unreliable readings [24]. Finally, as wearable sensors provide a measure for surface temperature, challenges arise for estimation of the core temperature [25], leading to unavoidable inaccuracy and drawbacks for application in clinical setting.

### 2.2. Novel Materials for Heart Rate Measurement

Monitoring of heart rate is an important measure for health of the cardiovascular system and also the whole body. As pulse rate is a direct mirror of heart rate in this review we consider them as the same. Generally, a comprehensive neuronal and hormonal system controls heart rate depending on the activity level of the body. Therefore changes in heart rate can happen as a response to different physiological and pathological conditions. For example physiological tachycardia during a cardio exercise causes a rise in heart rate very similar to tachycardia caused by thyrotoxicosis [26]. On the other hand, it has been recently observed that heart rhythm is not homogenous if we measure it over longer periods of time and it has periods of complex changes and stabilities depending on physical and mental conditions [27,28]. Therefore long-term monitoring of heart rate (HR) and heart rate variability (HRV) has important clinical potentials.

**Figure 2:**
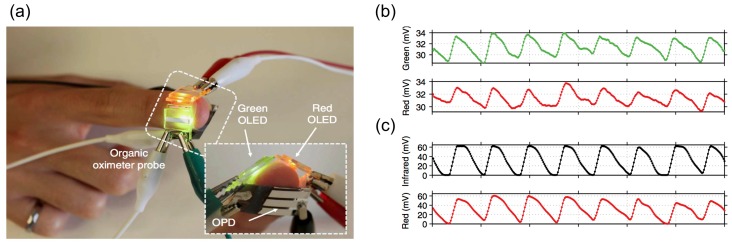
Flexible pulse oximeter device made from organic light-emitting diodes (OLEDs) and organic photodiode (OPD): (**a**) placement of red and green OLED on the subject finger with OPD placed on the opposite side; (**b**) heart rate detected by flexible setting; and (**c**) heart rate detected by conventional pulse oximeter device (Reprinted with permission [9]. Copyright 2014, Nature Publishing Group).

Commercially available wearable devices, such as Fitbit and Apple Watch use an optical modality called plethysmography to obtain heart rate. In this method a light emitting diode (LED) is placed in close contact to skin, which shines light on arteries. After absorption and scattering of light from the tissue, the reflected light is detected by photodiodes. Because the oxygenated hemoglobin has the highest light absorption, minimum intensities of reflected light are observed during systole. Considering that the measured reflection can be modulated by ambient light, motion and contact quality, complex algorithms are used to extract heart rate. Although the optical method is established for measuring heart rate and blood oxygen saturation (SpO_2_) in critical care unit and emergency wards, for long-term heart rate monitoring with wearable devices, it is subjected to challenges for motion induced accuracy, contact quality and high power consumption. The most common method for decreasing power consumption of LED/detector system for longer term use include reducing the duty cycle of the system by using fast LEDs and photodetectors that capture data in short span of time [29], which creates a trade-off between power consumption, accuracy and motion sensitivity. While current LED and photodetector HR sensors are rigidly packaged together, there is interest to develop flexible optical HR sensors that can provide better contact to skin and improved design forms. Lochner et al. [9] have recently reported a flexible pulse oximeter patch made from organic light-emitting diodes (OLEDs) and organic photodiode (OPD) (see Figure 2a). They have demonstrated heart rate estimation for this flexible patch with similar sensitivity as commercially available pulse oximeters (Figure 2b,c).

**Figure 3:**
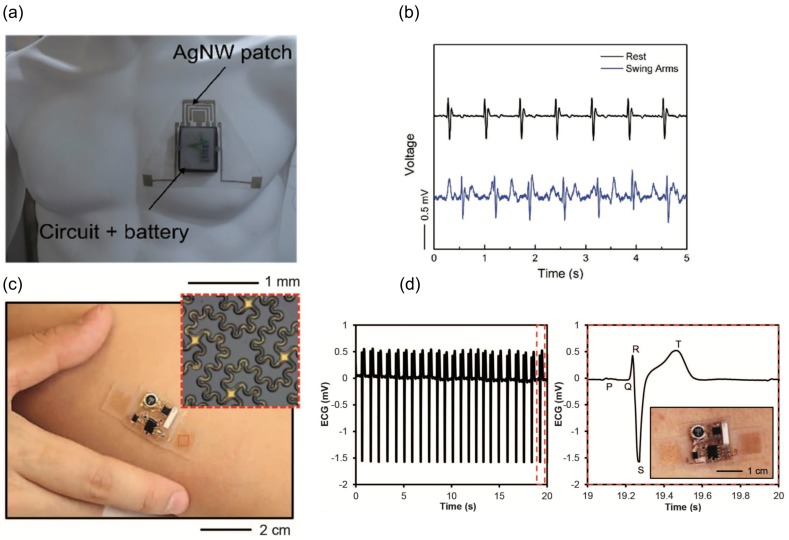
Wearable electrocardiography (ECG) monitoring devices: (**a**) integrated chest patch for ECG and skin impedance monitoring by electrodes made from AgNW encapsulated in PDMS (Polydimethylsiloxane); and (**b**) recorded ECG in resting and swinging arms conditions (Reprinted with permission [11]. Copyright 2017, John Wiley and Sons); (**c**) Flexible and stretchable ECG sensing device attached to forearm; and (**d**) acquired ECG by placing the device on sternum (Reprinted with permission [12]. Copyright 2014, The American Association for the Advancement of Science).

Another method that has been tried by many groups for wearable monitoring of heart rate is electrocardiography (ECG). Although the gold standard in cardiology is to use 12 leads ECG, one ECG lead can provide heart rate and may be used to detect of some cardiac pathological conditions such as arrhythmia. An approach of implementation of ECG monitor is to place two electrodes on the front of the chest so as to get a lead I ECG. While conventional electrodes are often rigid Ag/AgCl and for improved contact a gel is applied, there is interest in developing flexible and dry electrodes. Nemati et al. [10] incorporated small capacitive electrodes into a cotton T-shirt along with the necessary signal processing and communication circuits. Yao et al. [11] reported a chest patch with AgNW (Ag nanowire) /PDMS (Polydimethylsiloxane, Sylgard 184) dry electrodes (see Figure 3a,b), which can monitor Lead I ECG as well as skin impedance. Another approach is to design electrodes in a form of a band, which can be attached to forearm so as to provide one lead ECG. Xu et al. [12] incorporated two electrodes in a flexible band alongside with other necessary circuits to monitor ECG (Figure 3c,d). The main drawback of ECG for wearable HR monitoring is the high susceptibility of this modality to motion artifacts, especially because of the necessity of using dry electrodes for wearable devices.

**Figure 4:**
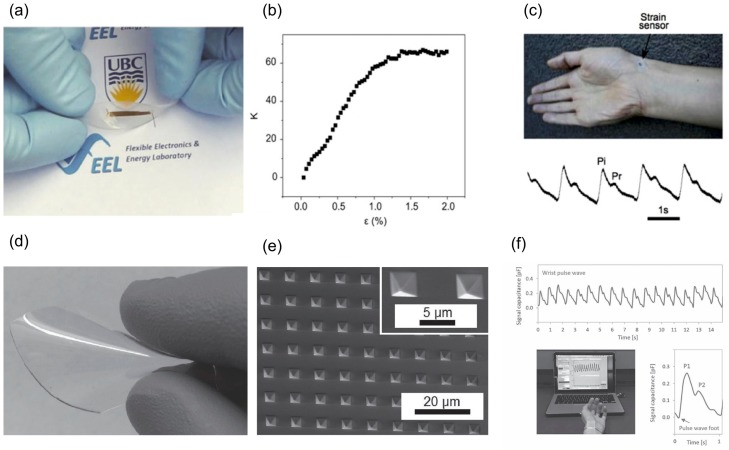
Application of strain and pressure sensors for heart rate monitoring: (**a**) flexible piezoresistive strain sensor fabricated by electrospinning and encapsulated in PDMS; (**b**) exceptional gauge factor of 60; and (**c**) detection of heart rate and pulse waveform by attaching the sensor on radial artery (Reprinted with permission [13]. Copyright 2014, Cambridge University Press); (**d**) flexible pressure sensing film; and (**e**) array of micro-pyramid shape structures fabricated on poly(glycerol sebacate) (PGS); (**f**) Blood pulse wave of the radial artery detected by the pressure sensor (Reprinted with permission [14]. Copyright 2015, John Wiley and Sons).

Another modality for HR monitoring which is coming to focus in the recent years is the application of flexible strain or pressure sensors. Soltanian et al. [13] reported a soft and conformable strain sensor made from a mesh of conductive nanofiber (NF) encapsulated in an elastomer (Figure 4a). Flexible piezoresistive sensors (FPS), which are manufactured using electrospinning—a versatile method for economic nanomaterial fabrication—demonstrates a fast, low-voltage, accurate, repeatable strain sensing behavior with a strain gauge factor as high as 60 (Figure 4b). Low power sensor operation down to μW will be feasible using this sensor [13]. The strain-sensitivity is attributed to the reversible disjointing/jointing of inter-NF junctions held together by a spring action of the encapsulating elastomer. By placing strain sensor on the radial artery, they reported an accurate pulse rate alongside a clear pulse waveform, from which it is possible to calculate even blood pressure estimate by pulse-wave velocity (PWV) measurement (Figure 4c). Pressure sensors also reported for heart rate monitoring purposes. Boutry et al. [14] reported a biodegradable pressure sensor consist of a flexible capacitor laminated between two layers of flexible substrates. An array of micro-pyramid shape structures was fabricated on poly(glycerol sebacate) (PGS), which can elastically deform by external pressure (Figure 4d,e). By placing the sensors on different arteries such as radial, femoral and carotid, they showed the feasibility of real-time HR and pulse waveform monitoring (Figure 4f). Luo et al. [30] also demonstrated a wearable sensor patch that integrates FPS with ECG electrodes for monitoring of heart rate and estimating the blood pressure. The FPS sensors made by integration of a layer of carbon-decorated fabric with Au interdigitated electrodes, both encapsulated with polyethylene naphthalate (PEN). The piezoresistive behavior of the device originated from the variation in the contact among the carbon particles and the electrode underneath during press and release cycles.

### 2.3. Respiratory Monitoring

Respiratory rate is one of the most important vital sign and changes in respiratory rate are important indicator of major physiological and pathological instabilities. A change in respiratory rate of a subject is a very good indication of cardiopulmonary illnesses such as acute respiratory syndrome (ARDS), chronic obstructive pulmonary disease (COPD), pulmonary edema, pulmonary emboli, pneumonia, heart failure and many other conditions [31]. In addition, it has been established that changes in respiratory rate can be used to detect serious clinical conditions such as cardiac arrest and requirement for admittance to intensive care unit [32,33]. On the other hand, measurement of respiratory function and rate from different body locations can have important clinical significance. For instance, thoraco-abdominal asynchrony, non-coincident motion of rib cage and abdomen during breathing, is an important sign of respiratory distress [34]. Interestingly, despite the importance of respiratory monitoring, it is considered to be the most neglected vital sign in clinical set up [35,36]. One of the main reasons for this problem is the lack of available systems for continuous and accurate measurement for clinical application, which means huge opportunity for novel sensors to be developed.

**Figure 5:**
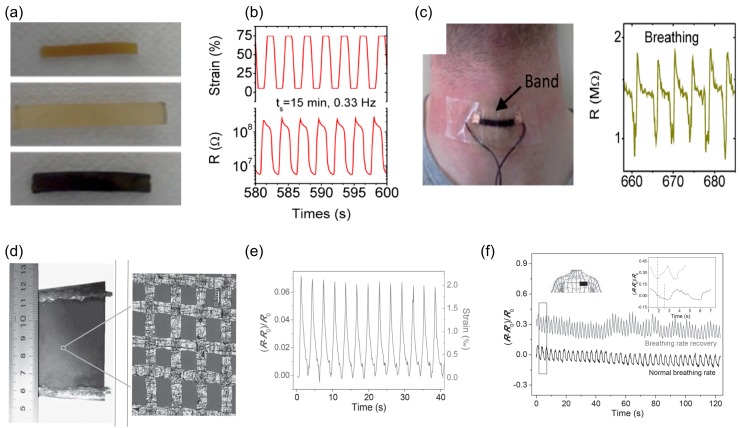
Strain sensors for respiratory monitoring: (**a**) treatment of rubber band to infuse graphene, top to bottom, respectively, show untreated elastic band, elastic band after soaking in toluene for 3.5 h and a graphene-infused band; (**b**) resistance changes of sensors as a response to cyclic strain and release; and (**c**) graphene infused band attached to the throat to monitor respiratory rate (Reprinted with permission [15]. Copyright 2014, American Chemical Society, http://pubs.acs.org/doi/full/10.1021/nn503454h); (**d**) Optical images of graphene woven fabric deposited on PDMS composite film; (**e**) relative resistance changes between 0% and 0.2% strain; and (**f**) schematic picture of placement of the sensor on chest and the respiratory rate detected by it normally and while recovering from exercise (Reprinted with permission [16]. Copyright 2014, John Wiley and Sons).

Methods for respiratory monitoring could be divided into noncontact and contact methods. Non-contact methods such as infrared thermography [37], radar based methods [38] and optical methods [39] have the advantage of not needing a direct contact with the patient, but suffer from difficulty of usage and inaccuracy [40]. On the other hand, contact based methods can provide more accurate results by using wearable sensors, and therefore fit better in the focus of this paper. One of the established methods for continuous respiratory rate monitoring, especially in post-anesthesia care unit (PACU), is to place acoustic sensors on airways, which provide accurate data but is susceptible to movement [41]. An important advantage of acoustic sensors is the ability to detect airway obstruction in which chest wall is moving but there is no airflow into the lungs [42]. Another approach is to use strain or pressure sensors made from novel materials. Boland et al. [15] reported a conductive composite made from graphene infused rubber with high strain sensitivity (strain gauge of up to 35) and working at strains exceeding 800% (Figure 5a,b). By attaching a piece of encapsulated rubber composite to the subject’s throat, they reported respiratory rate accurately (Figure 5c). Wang et al. [16] demonstrated strain sensors based on graphene woven fabric (Figure 5d,e) for respiratory rate monitoring (Figure 5f).

**Figure 6:**
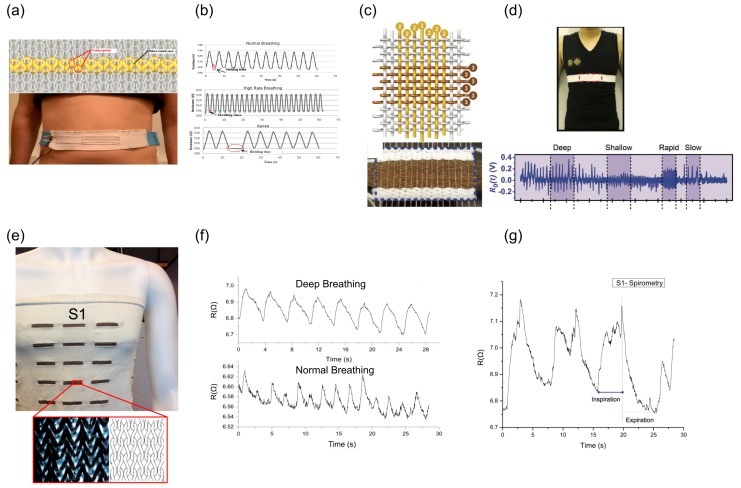
Textile based sensors for respiratory monitoring: (**a**) schematic diagram (top) and the actual prototype of chest strap made from conductive silver-coated yarn (yellow) woven into normal yarn; and (**b**) monitoring of respiratory rate in different scenarios such as normal, high-rate and apnea (Reprinted with permission [15]. Copyright 2014, American Chemical Society); (**c**) Schematic of warp and weft of Cu coated yarns (top) and as-woven smart textile (bottom); and (**d**) placement of the chest strap made from the smart textile (top) and raw signals detected during breathing (Reprinted with permission [18]. Copyright 2016, John Wiley and Sons); (**e**) Ag plated yarn integrated into a T-shirt (top) the fabric structure (bottom), for the purpose of monitoring only S1 activated while it is possible to activate all the sensors to get an array of responses; (**f**) normal breathing and deep breathing detected by the smart T-shirt; and (**g**) response of the smart T-shirt during spirometry type experiment showing the gradual chest inflation followed by rapid deflation (Reprinted with permission [44]. Copyright 2014, SAGE Publications).

Textile based sensing is another important approach which provides a more conformable and user-friendly approach for respiratory monitoring. These types of sensors are mainly based on application of conductive yarn or fiber, which is integrated into textiles by different methods. Atalay et al. [17] have used knitted silver coated nylon yarn to make a respiratory belt, which can be worn around the chest or abdomen to monitor respiratory rate (Figure 6a,b). Zhao et al. [18] also reported fabricated smart textile made by weaving of Cu-coated polyethylene terephthalate (Cu-PET) warp yarns and polyimide (PI)-coated Cu-PET (PI-Cu-PET) weft yarns. They also made a chest strap that can monitor not only the respiratory rate but also can detect changes in respiration patterns (Figure 6c,d). Wang et al. [43,44] reported a comprehensive model and experimental results for totally integrated conductive knitted fabric based on a loops structure under biaxial extensions. The fully integrated strain sensor is knitted into a T-shirt (Figure 6e) and has been tried to show it can detect both respiratory rate and changes in pattern such as deep breathing compared to normal breathing (Figure 6f). On the other hand we have also tested it in spirometer type of breathing, which consisted of a deep inspiration followed by a rapid expiration, in which the deflation pattern of the chest wall has clinical significance, especially during the early stages of rapid expiration (Figure 6g).

## 3. Activity, Posture and Muscle Movement Monitoring

Monitoring muscle movements, posture and body activity is another important aspect of wearable devices for wellness, fitness and clinical applications. Recently, consumer devices, such as Fitbit One^®^ and Fitbit Flex^®^, have been tested and examined to monitor patients step counts, energy expenditure and activity levels [45]. The results from reliability and validity examinations have shown promising future for wearable activity monitoring in many clinical settings such as post-surgery recovery in cardiac patients, pulmonary rehabilitation, activity counseling in diabetic patients and assessment of chemotherapy candidates [46,47]. In addition to activity monitoring such as measuring number of steps and estimating energy consumption, wearable sensors can play an important role in localized monitoring of muscle movement, muscle function and body posture in real-time.

Current activity monitoring devices rely on inertial measurement units (IMUs) including accelerometers and gyroscopes for estimating the three degrees of freedom (3 DOF) translational and 3 DOF rotational (angular) movements in space and correlating it to body or limb movements and activities. IMU sensors with 9 DOF that include magnetometer (compass) or 10 DOF with barometer can be used to provide more detailed movement information. For activity tracking, a single 6 DOF IMU sensor with complex feature extraction and motion separation algorithms are necessary to detect different movements and identify activities. More complex motion tracking, such as detection of limb and body part movements and posture is possible through securing multiple IMUs with important clinical applications from lower back pain [48] to stroke rehabilitation [49] and gait monitoring [50]. For example, Hajibozorgi et al. [48] have used multiple IMUs placed on the back to monitor posture and range of motion (ROM) of spine. An important drawback of the use of IMUs is the need for multiple devices at different locations of the body and complex algorithm and data processing for cross-referencing the anatomical landmarks as well as the sensitivity of the data to motion noise, looseness of orientation and point of contact with body and rigidity, bulkiness and high power consumption of the sensor nodes. The power consumption of the sensors (mW to 10 s of MWs) [51] can be reduced by reducing duty cycle and sampling rates, signifying a trade-off between power consumption, accuracy, and noise.

**Figure 7:**
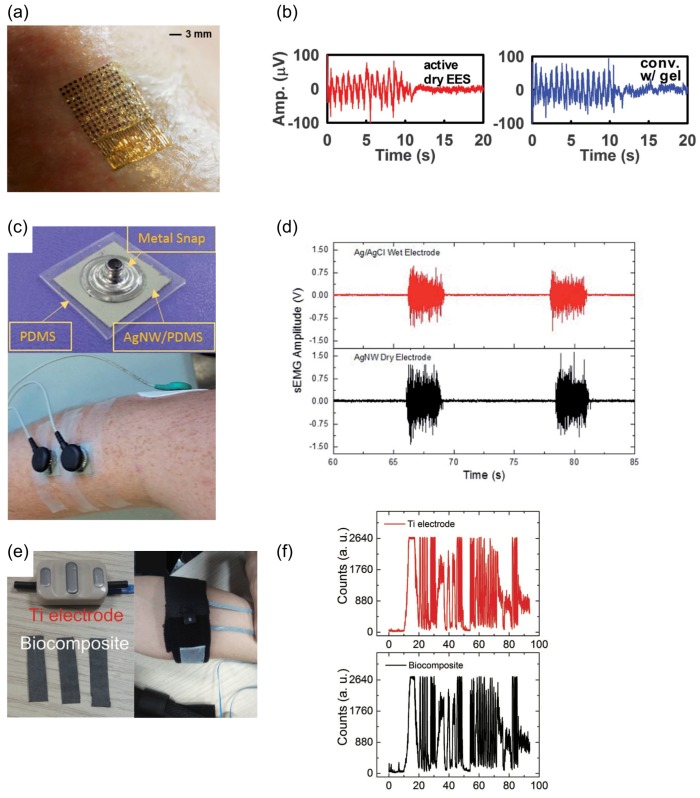
Wearable sensors for electromyography (EMG) monitoring: (**a**) image of an epidermal electronic sensor (EES) attached to skin; and (**b**) EMG measurements using an active EES mounted on the subject leg alongside with a conventional EMG electrode with similar detected signals (Reprinted with permission [53]. Copyright 2014, The American Association for the Advancement of Science); (**c**) AgNW dry electrodes and their placement on the subject’s leg muscle, in parallel with conventional wet-Ag/AgCl electrodes; and (**d**) EMG data recorded by wet-Ag/AgCl electrodes and AgNW dry electrodes (Reprinted with permission [54]. Copyright 2015, Royal Society of Chemistry); (**e**) Photographs showing standard Ti EMG electrodes in comparison with biocomposite on subject’s arm; and (**f**) recorded EMG signals from both electrodes (Reprinted with permission [55]. Copyright 2016, John Wiley and Sons).

On the other hand, localized monitoring of muscular movement and function has clinical significance in many different fields including neurophysiology, sport medicine, rehabilitation, occupational medicine, proctology, obstetrics, gait analysis and physical therapy [52]. The established method for this purpose is electromyography (EMG) in which surface electrodes record the electric activity of the muscles beneath the skin. Recently, advances in flexible electronics and nanomaterial have opened huge opportunity for development of conformable EMG monitoring using novel materials. Kim et al. [53] reported development of epidermal electronic system (EES), which leads to conformal contact and adequate adhesion based on van der Waals interactions (Figure 7a). Using this type of EES materials they demonstrated EMG signals from different locations including leg and neck, with a sensitivity similar to that of a commercially available system (Figure 7b). Myers et al. [54] presented a silver nanowire (AgNW) based dry electrode for electrophysiological wearable monitoring. The AgNWs are inlaid below a thin layer of PDMS (Figure 7c), and the encapsulated electrodes is attached to the forearm to detect EMG, the response detected from this type of electrodes are similar to that detected using a conventional Ag/AgCl wet electrode (Figure 7d). Cataldi et al. [55] demonstrated cellulosic graphene biocomposites electrodes made by infusion of graphene nano-platelet into cellulose fibers (Figure 7e) and sensitive to detect EMG from forearm similar to commercially available titanium (Ti) electrodes (Figure 7f).

**Figure 8:**
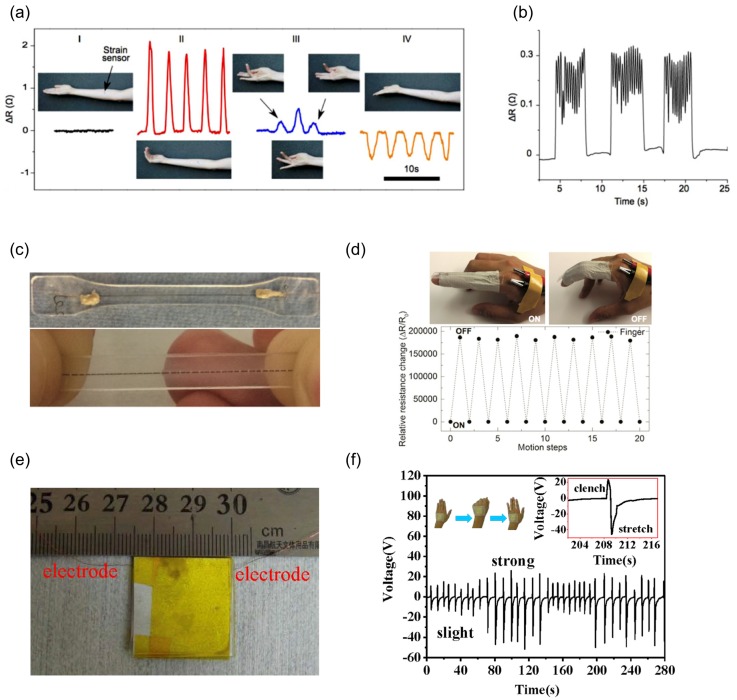
Sensors for non-invasive muscle and joint monitoring. (**a**) Application of nanofiber sensor attached to epidermis of forearm flexor bundle monitoring hand gestures: (I) neutral hand position; (II) repeated wrist flexion; (III) subsequent flexion of the second, third and fourth digits; and (IV) repeated wrist extension; (**b**) Accurate monitoring of simulated intermittent Parkinson’s tremors by nanofiber sensor attached to subject’s forearm (Reprinted with permission [13]. Copyright 2014, Cambridge University Press); (**c**) Photographs from a typical single-wall carbon nanotube (SWCNT) wire embedded in PDMS (top) and the cracks happening in the wire caused by stretch (bottom); (**d**) CNT wire sensor mounted on fingers detecting finger flexion and extensions (Reprinted with permission [56]. Copyright 2016, Royal Society of Chemistry); (**e**) Photograph of the as prepared triboelectric sensor; (**f**) Detection of hand movements by attaching a T-sensor to the dorsal side of the hand (Reprinted with permission [57]. Copyright 2016, Elsevier).

Other groups have shown use of strain sensors for detection of muscle contraction and expansion. For example, Soltanian and Servati et al. [13] attached a nanofiber based strain sensor to the flexor bundle of the forearm to monitor flexion and extension movements of the wrist and fingers (Figure 8a). Having the same sensor on the flexor bundle they reported accurate monitoring of simulated intermittent Parkinson’s tremors (Figure 8b). Zhou et al. [56] demonstrated a deformation sensor made from wet-spun single walled carbon nanotube (CNT) in PDMS Figure 8c. The CNT wire gets fragmented under strain causing an increase in its resistance. Using this type of novel sensors they showed it is possible to detect joint movements (Figure 8d). Using a different approach, Cai et al. [57] reported a flexible sensor based on triboelectric effect, induction of electrostatic charges on the surfaces between two different materials. Their sensors were made from a layer of roughed polyethylene terephthalate aluminum (R-PET-Al) and a layer of polytetrafluoroethylene (PTFE) film. Using this type of sensor, they monitored not only muscle movement but also other vital signs (Figure 8).

Modern life style put body under pressure because of awkward postures required during occupational and habitual activities. Therefore, prevalence of problems caused by body posture radically increased, as an instance the lifetime prevalence of low back pain (LBP) is as high as 60 to 80% [58]. To overcome this important public health issue, there is need for real-time monitoring devices for diagnosis, rehabilitation and biofeedback applications. Novel wearable sensors could play an important role in this field. Different modalities have been tried for posture monitoring including optical sensors [59], accelerometers [60], gyroscope [61] and strain gauge sensors [62]. Novel flexible materials and sensors can enable continuous and real-time posture monitoring. O’Sullivan et al. [63] tested the validity and reliability of a commercially available device for spinal posture monitoring called “BodyGuard” and showed that strain gauge sensors can monitor sagittal spinal posture and its range of motion with similar sensitivity of optical method, which means novel flexible strain sensors could be the future for wearable posture monitoring.

## 4. Signal Processing for Wearable Sensors

Wearable sensors are prone to limited sensitivity and reliability because non-invasive monitoring of many clinical parameters basically means weak signals that can be masked by motion and other artifacts. Therefore, suitable data processing methods are needed to analyze data collected from wearable sensors for personalized applications. Considerable efforts have been made to address the following data processing challenges: data acquisition and artifacts removal, sensor data mining and sensor fusion.

### 4.1. Data Acquisition and Artifacts Removal

The first stage of wearable monitoring is data acquisition, which aims to collect physiological signals and movement data via wearable sensors. As introduced before, wearable sensors measure different types of physiological signals (such as photoplethysmogram (PPG), partial oxygen blood saturation (SpO_2_), skin temperature, etc.) and physical activity signals (such as acceleration, position, etc.). While these data can be of various types, the corresponding sensors always are attached to subjects’ skin at given places of the body. Then the change of the signal of interest can be reflected by the change of sensors’ electrical properties. For instance, Selvam et al. [64] monitored alcohol consumption lifestyle via ethyl glucuronide detection in human sweat based on wearable biochemical sensor. The impedance change was measured to characterize the chemiresistive interactions occurring on the sensor surface. Many wearable monitoring systems also involve integrated amplifiers for the reason that the acquired signal is too tiny. Then the analog-to-digital converter digitizes the signal before further analysis.

Prior to conduct any other signal processing task, it is essential to remove the unwanted signal disturbances. The acquired signals may be contaminated by different types of noise and distortions, such as instrumentation noise, motion artifacts and other types of interference. The suitability of specific artifact removal techniques greatly depends on the application and the nature of the sensor signals (e.g., the data statistics, the stationarity of the desired signal and the noise).

Artifacts removal techniques usually involve a filtering process, implemented in the hardware or later in a digital software manner. Filtering is often used to remove high or low frequency noise and the main power interference, typically by limiting the bandwidth of the measured signals to the band of interest, by notching unwanted frequencies. However, commonly used filtering techniques, such as bandpass filter, fail to remove the artifacts when the desired signals and the artifacts overlap in the frequency domain or when the noise has a non-stationary nature [65]. To overcome this challenge, adaptive de-noising strategy attempts to cancel out unwanted artifacts correlated with the reference signal which itself is provided by another sensor/noise generator [66,67]. For instance, Kim et al. utilized accelerometers as noise reference signals to remove the motion artifact from the PPG signal [66]. In order to ensure the high correlation between the reference signals and the unwanted artifacts, Yousefi et al. took into account the motion artifact due to various sources and proposed a reference noise generator. They further developed a two-stage Normalized Least Mean Square (NLMS) adaptive noise canceler [67]. In consideration of the non-stationary nature of the physiological signals, wavelet transform and empirical mode decomposition (EMD) can be employed to remove the artifacts [68,69]. Another approach is based on blind source separation, which utilizes specific statistical properties of the acquired signals to separate the desired source signals from unwanted artifacts [70,71,72]. Salehizadeh et al. proposed a novel motion artifact reduction technique based on singular spectrum analysis (SSA) [72]. Considering the underdetermined scenario, Zou et al. combined noise assisted multivariate empirical mode decomposition (NAMEMD) and multiset canonical correlation analysis (MCCA) to recover the heart beat signal from three Nanofiber-based strain sensors [73]. For more information about artifact removal techniques, we refer the interested readers to comprehensive reviews provided by Celka et al. [74] and Tamura et al. [75].

### 4.2. Wearable Sensor Data Mining

Employing different data mining techniques, wearable sensors can be utilized to identify critical conditions—such as heart attack and falling—to alert the users or their family members, as well as monitoring the state of the users, in different situations. The first stage of data mining consists of feature extraction. It provides a meaningful and compact representation of the time series signal. This stage can be achieved by considering the statistical property of the time series signals or knowledge from the problem-specific domain. Statistical features, including features from time domain and frequency domain, can be extracted from the raw signals independently of its application. In the time domain, the extracted features usually contain basic waveform characteristics and statistical parameters, such as mean value, variance, skewness and kurtosis. In addition, a great deal attention has been paid to extract features in the frequency domain, such as power spectral density, energy and wavelet coefficients. The extraction of application-specific features should also take into account the domain knowledge, such as fiducial points of PPG signals and specific iris features [76,77]. For instance, Gu et al. proposed a novel human verification approach based on four parameters from PPG signals from 17 subjects, including the peak number, time interval, upward and downward slope [76]. Feature extraction attempts to reduce the dimensionality of the time series signals and alleviate the effect of noise. However, the extracted features themselves can also introduce noise and some of them could be redundant or misleading. Therefore, at the second stage, it is desirable to select the best subset candidate features before further analysis based on certain characteristics of all extracted features. For instance, Mao et al. extracted 34 features from heartbeat and oxygen saturation signals. They adopted a forward feature selection algorithm and selected relevant features to enhance the accuracy of deterioration warning [78]. Besides the quality of the acquired signals and the extracted/selected features, the performance of the wearable sensor system also depends on machine learning algorithms. Each algorithm has its own advantages and limitations. For instance, the algorithm of K-Nearest Neighbors (KNN) is robust to noisy training data and effective when the training data is large. However, we need to determine the value of K and choose the suitable type of distance to get the best performance. In addition, time to find the nearest neighbors in a large training set can be excessive. Therefore, it is not suitable for the wearable monitoring system with limited computational ability. Support vector machines (SVMs) always provide high accuracy, however they are memory intensive and are not recommended for small memory system. The criterion to choose either of these methods is subject to the nature of the acquired signals and the application of interest. Table 2 lists some existing wearable sensor systems and some utilized machine learning techniques.

### 4.3. Sensor Fusion

Many real-world applications involve multiple sensors whose data need to be jointly analyzed [88,89]. Wearable health/activity monitoring systems relying on a single sensor could suffer from several limitations, such as limited spatial coverage and the system/data uncertainties. Generally, combining various wearable sensor signals can yield better recognition performance. An effective way to combine multiple sensor data is represented as sensor fusion. The fusion can be performed at any stage of the signal processing and decision-making process including the raw data-level, feature-level, and decision-level. Different sensor fusion methods can be adopted depending on the specific problem and the collected signals. If the sensors measure the same type of physical phenomena, the acquired signals from these sensors can be directly fused. A good example of this approach is the fusion of three-axis accelerometer data for gait recognition [90]. Otherwise, data generated from heterogeneous sources cannot be combined directly and should be fused at the feature extraction stage or decision-making stage. In feature-level fusion, features extracted from multiple sensors separately can be concatenated into a new high dimensional feature as the input of the further classification/pattern recognition step. A classic example is the fusion of accelerometer, magnetometer, and gyroscope data for biometric applications [91]. Decision-level fusion is the process of combining the decisions generated from multiple sensors individually [92]. Depending on the confidence of each classifier/decision machine, decisions can be fused in a weighted voting manner. For more information, please refer to [93,94,95] and the references therein.

## 5. Conclusions

Wearable health monitoring for clinical applications is a rapidly emerging field that can improve quality and accessibility of health care. Novel materials can play an important role in solving technical issues such as high power consumption, lack of accuracy, limited reliability and repeatability. In addition, as technologies move toward smart textile, this can also improve the conformability problem of the sensors and therefore can be used seamlessly with limited interruption in daily life activities, while providing crucial information. In this review paper, we reported novel technologies applied for wearable monitoring of vital signs, including body temperature, heart rate and respiratory rate, followed by advances in the field of muscle and activity monitoring. For each of these clinically important parameters, there are different modalities and platform technologies available, which have been discussed and compared with novel technologies. This review also tries to show the drawbacks, limitations and opportunities available for successful translation of recent technological advances for clinical applications. Finally, in the final section of this paper, new advances in the field of signal processing for wearable health monitoring purposes have been described thoroughly, which is an important part of the development of sensors and wearable devices with clinical significance.

## Figures and Tables

**Table 1:** Examples of novel wearable sensors and associated materials for vital sign monitoring.

Reference	Vital Signs	Sensor Materials	Key Features	Limitation
[5]	Temperature (T)	Graphene	Flexible and stretchable	Sensitivity fluctuation under Strain
[6,7]	T	Printed NiO	High Sensitivity, scalable manufacturing	Not stretchable
[8]	T	Organic semiconductors (BEDT-TTF)	Flexible	Unknown sensitivity and robustness
[9]	SpO2/Heart rate (HR)	Organic semiconductors for OLED and OPD	Highly flexible	Requires tight contact
[10,11,12]	ECG/HR	Textile, AgNW/PDMS and printed electrodes	Accurate and clinically relevant	Limited form factor
[13]	HR	Piezoresistive nanofibers	Highly sensitive, flexible and potential for BP	Prone to motion artifact
[14]	HR	PGS based pressure sensor	Potential for BP	Required tight contact
[15,16]	Respiratory Rate (RR)	Graphene infused rubber, graphene woven fabric	Highly stretchable, scalable manufacturing	Cost and prone to motion artifacts
[17,18]	RR	Silver coated and PI-Cu-PET coated yarn	Textile based	Low sensitivity

**Table 2:** Some existing wearable sensor systems and their related machine learning techniques and applications.

Reference	Sensors	Extracted Features	Machine Learning Method	Aim
[79]	Accelerometer	Discrete Cosine Transform	K-Nearest Neighbors (KNN)	Activity recognition
[80]	Collar-integrated electrodes	Time domain features	Linear Discriminant Analysis (LDA)	Upper body activity recognition
[81]	Accelerometer	Acceleration time series	Hidden Markov Model (HMM)	Fall detection
[82]	Bioimpedance sensor	Bioimpedance magnitude and phase	Naive Bayes (NB)	Biometrics
[83]	Pressure and acceleration sensor	Pressure and acceleration histories	Support Vector Machine (SVM)	Activity recognition
[84]	Accelerometers	Time domain features	SVM	Estimation of metabolic equivalent of tasks
[85]	Electrocardiogram	Clinical features	HMM	Cardiac arrhythmias
[86]	Electromyography, respiration and Other sensors	Time and frequency domain features	SVM	Emotion evaluation
[87]	Accelerometers	NA	Deep convolutional and LSTM recurrent neural networks	Activity recognition
